# CusProSe: a customizable protein annotation software with an application to the prediction of fungal secondary metabolism genes

**DOI:** 10.1038/s41598-023-27813-y

**Published:** 2023-01-25

**Authors:** Leonor Oliveira, Nicolas Chevrollier, Jean-Felix Dallery, Richard J. O’Connell, Marc-Henri Lebrun, Muriel Viaud, Olivier Lespinet

**Affiliations:** 1 Institute for Integrative Biology of the Cell (I2BC), Université Paris-Saclay, CEA, CNRS, 91198 Gif-sur-Yvette, France; 2 Université Paris-Saclay, INRAE, UR BIOGER, 78850 Thiverval-Grignon, France; 3 Present Address: Orphanet-INSERM, US14, Plateforme des Maladies Rares, Paris, France

**Keywords:** Computational biology and bioinformatics, Microbiology

## Abstract

We report here a new application, CustomProteinSearch (CusProSe), whose purpose is to help users to search for proteins of interest based on their domain composition. The application is customizable. It consists of two independent tools, IterHMMBuild and ProSeCDA. IterHMMBuild allows the iterative construction of Hidden Markov Model (HMM) profiles for conserved domains of selected protein sequences, while ProSeCDA scans a proteome of interest against an HMM profile database, and annotates identified proteins using user-defined rules. CusProSe was successfully used to identify, in fungal genomes, genes encoding key enzyme families involved in secondary metabolism, such as polyketide synthases (PKS), non-ribosomal peptide synthetases (NRPS), hybrid PKS-NRPS and dimethylallyl tryptophan synthases (DMATS), as well as to characterize distinct terpene synthases (TS) sub-families. The highly configurable characteristics of this application makes it a generic tool, which allows the user to refine the function of predicted proteins, to extend detection to new enzymes families, and may also be applied to biological systems other than fungi and to other proteins than those involved in secondary metabolism.

## Introduction

Fungal secondary metabolites (SM), also known as specialized metabolites, are an important source of compounds of pharmaceutical and agrochemical interest. These include antibiotics, immunosuppressants, and phytotoxins with a wide range of molecular targets^1,2^. Although fungi have been exploited for decades for their potential in antibiotic and pharmaceutical production, the chemical diversity of fungal SM and their potential biological activities remain under-explored. Indeed, the analysis of fungal genomes has revealed the presence of huge repertoires of genes involved in the biosynthesis of SM, indicating that these organisms have the capacity to produce many more compounds than those described to date^2^. In particular, the genome analysis of plant and insect pathogenic fungi has revealed their potential to produce a wide range of previously uncharacterized compounds^3–5^.

The genes encoding enzymes involved in the biosynthetic pathways producing secondary metabolites fall into two categories: genes encoding key enzymes and genes encoding accessory enzymes. Key enzymes are involved in the essential step of the biosynthetic pathway, usually the first step in this pathway that leads to the synthesis of the metabolite skeleton^6^. In the absence of this enzyme, the final metabolite is not produced. The main families of fungal SM key enzymes (SMKEs) are (i) polyketide synthases (PKS), (ii) non-ribosomal peptide synthetases (NRPS), (iii) hybrid PKS-NRPS, (iv) dimethylallyl tryptophan synthases (DMATS), and (v) terpene synthases (TS)^6,7^. Accessory enzymes act upstream or downstream of the essential stage of the biosynthetic pathway, either producing precursors used by the key enzyme or modifying the metabolite produced. Most frequently accessory enzymes are glycosyl transferases, methyltransferases, reductases and oxidases, particularly cytochromes P450 oxidoreductases^6^. The genes encoding key and accessory enzymes of a given SM pathway are usually physically linked into a gene cluster with a shared transcriptional control^8,9^. The annotation of secondary metabolism genes in fungal genomes is of great interest for the discovery of new bioactive compounds and for the understanding of their biosynthesis.

In the past years, several computational methods have been developed to help researchers in mining microorganism genomes for SM genes and clusters, and multiple reviews have been published comparing the algorithmic logic behind each software, as well as their advantages and limitations^10–13^. Classically, genome mining approaches focus on the identification of genes encoding SMKEs, based on their sequence conservation. Some of these tools are dedicated to specific classes of SMKEs, mostly PKS and/or NRPS^14,15^. Other software search for complete SM gene clusters. Examples are the “Secondary Metabolite Unknown Region Finder” (SMURF)^16^, a web-based tool for the mining of fungal genome sequences for PKS, NRPS, hybrid PKS-NRPS, and DMATS gene clusters, and “antibiotics and Secondary Metabolite Analysis SHell” (antiSMASH)^17,18^, for the identification of secondary metabolite biosynthetic gene clusters in fungal and bacterial genomes. These available genome mining tools are powerful and have led to the identification of new enzymes, compounds, and elucidation of biosynthetic pathways. However, some caveats still exist. One important limitation is the fact that most genome mining platforms use rule-based approaches in which pre-defined rules are implemented in the software. This means that only enzymes/pathways for which rules were previously established can be found.

In the present work, we developed a novel software, CusProSe, to assist users in mining genomes for proteins of interest based on their conserved functional domains. Unlike similar existing tools, CusProSe was designed to be a totally flexible workflow where the user can define not only their own protein families of interest but also the searching rules. Here, we used CusProSe for identifying SMKEs in phylogenetically diverged fungal species, as an example of application, and compared the results obtained with those from the existing predictors SMURF and antiSMASH.

## Results

### Development of the CusProSe software

**Figure 1:**
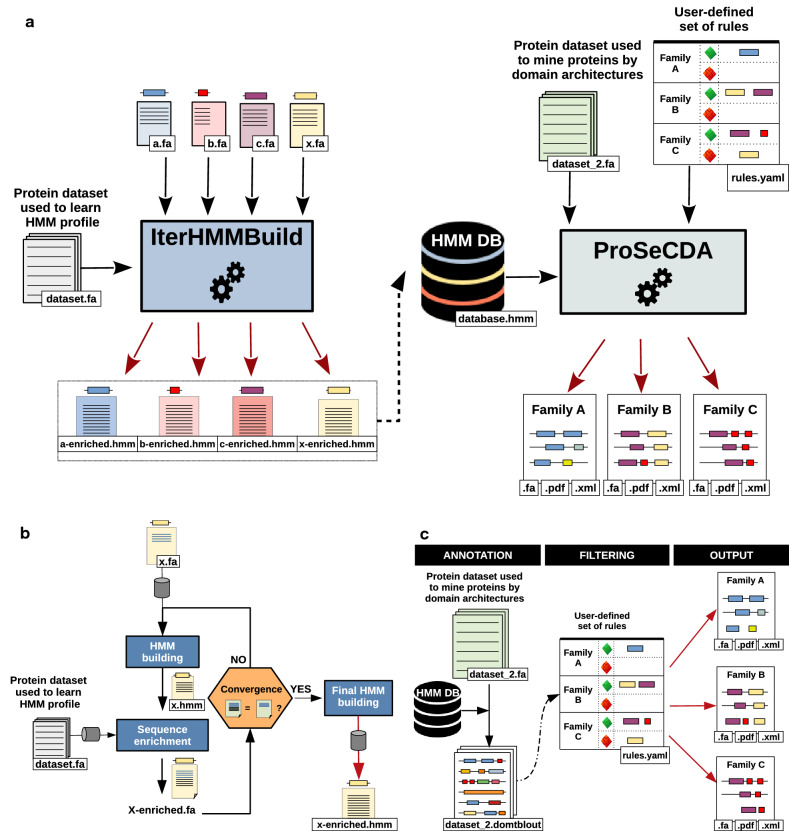
CusProSe package functionalities. (**a**) Overview of CusProSe. CusProSe contains two independent but complementary programs, IterHMMBuild and ProSeCDA. The figure schematizes the functioning of these tools. The IterHMMBuild program provides users with representative HMM protein profiles of interest, constructed by an iterative enrichment process, starting from a small set of defined protein seed sequences. Two inputs are required in a fasta file format: original seed sequence(s), (examplified here as a.fa b.fa c.fa and x.fa) and a set of other protein sequences such as a proteome (named here as dataset.fa) to iteratively feed the HMM profile. The output of iterHMMBuild includes, for each protein/protein domain of interest, the final HMM profile file (enriched.hmm). The different HMM profiles are then concatenated, and a database of profiles (database.hmm) is created and displayed in the output directory. ProSeCDA allows to search in a given protein dataset for multiple proteins of interest, defined by a user-specified set of domains and rules. The program takes as input a protein dataset of interest such as a proteome (dataset_2.fa file), an HMM profile database (database.hmm file) and a user-defined set of rules (rules.yaml file). The HMM profile database can the one created with IterHMMBuild or, alternative, any other user defined compatible database (in hmm format). (**b**) Overview of the IterHMMBuild iterative enrichement process. In the first step of the procedure an HMM profile model is build from the query protein sequences (x.hmm). This initial profile is then used to identify sequences with similar domains in the user-specified protein sequence dataset (dataset.fa). If matching sequences are found, they are added to the initial query sequence file (creating a new file named x-enriched.fa), and a new HMM profile is built. This process is repeated until no new sequences are recovered (i.e. convergence is reached). When convergence is reached a final HMM file is build (named here as x-enriched.hmm) and dispalyed in the output directory. (**c**) Overview of the ProSeCDA steps. The first step of the procedure consists in the annotation of the protein dataset of interest used to mine proteins (such as a proteome, named here as dataset_2.fa), with protein domains from a user-specified HMM profile database (HMM DB). In the next step, the annotated proteins (dataset_2.domtblout file) are filtered according to user-specified rules (rules.yaml file). Each rule is defined by different features including the protein family name, the “mandatory” list of domains (list of domains the protein must contain, green), and the “forbidden” list of domains (optional, list of domain the protein must not contain, red). All proteins matching those rules are selected and accessible in the output files. More details of Prosecda outputs are presented in Fig. 2.

CustomProteinSearch (CusProSe) is a generic genome mining software, consisting of two distinct but complementary customizable programs: IterHMMBuild and ProSeCDA. IterHMMBuild is an HMM profile building tool based on an iterative learning process. ProSeCDA is a protein search and annotation tool based on user-defined domain architectures. The two programs can be run independently. An overview of the CusProSe workflow and package functionalities is presented in Fig. 1. Detailed information about its implementation and functioning is provided in the “Methods” section as well as in the CusProSe documentation page (https://i2bc.github.io/CusProSe/).

**Figure 2:**
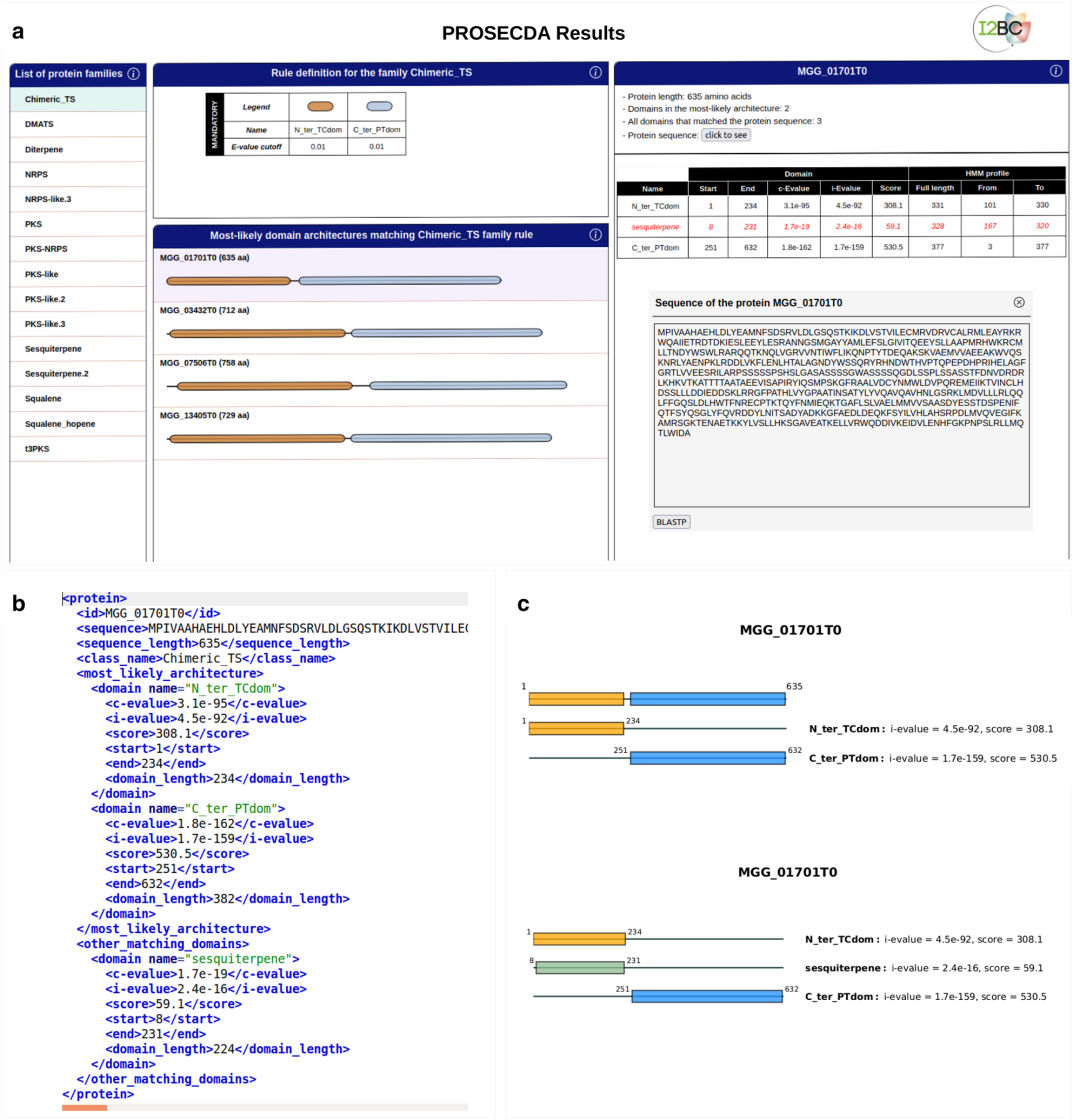
Output of ProSeCDA. (**a**) Interactive web page allowing to visualize the annotation results. The page displays different pannels. The user can get detailed information about each one by clicking on the “i” located on each pannel header, on the right. The left pannel is a list of the user-defined protein families for which proteins have been found. The user picks a protein family to visualize by clicking on the protein family name to select it, and update its related informations visible in the other pannels. (**b**) XML file showing details of an individual annotated protein. (**c**) Schematic visualization of the protein identified domains in pdf format (optional parameter of ProSeCDA). Two types of pdf files are generated: upper panel, a file containing graphical representations of the most-likely domain architecture of all the proteins matching the user-defined family rule. Only one protein is shown here as an example. Lower panel, a file for each individual protein representing all the domains that matched the protein sequence during the annotation step.

The CusProSe IterHMMBuild tool allows the users to construct HMM profiles representatives of their protein sequences of interest, by an iterative learning process starting from seed sequences and a fasta protein dataset (Fig. 1a,b). The IterHMMBuild procedure starts building an HMM profile from a set of related protein or protein domain seed sequences or from a single query sequence. This initial HMM profile is then used to identify sequences with similar domains in any user-specified protein sequence dataset, in order to enrich the profile model. If matching sequences are found, they are added to the initial query sequences and a new HMM profile model is built. This new HMM profile is then searched against the same target dataset in order to find more distant similar sequences to the original query sequence(s)^19^. This process is repeated (iterations) until convergence is reached, i.e., no new sequences are recovered from the dataset (Fig. 1b) (see also the supplementary Fig. S1 and “Methods” section for technical details). When convergence is reached a final HMM enriched profile is build. A database of HMM profiles is then created by concatenation of the individual final profiles, either automatically or manually depending on input parameters and the user’s choice. Additional information about the HMM database creation procedure can be found in the “Methods” section and in the Documentation page in the IterHMMBuild usage guideline chapter. As regards ProSeCDA, the tool allows to search in a given protein dataset, for multiple proteins of interest defined by a user-specified set of rules (Fig. 1a,c). The first step of the ProSeCDA pipeline is to annotate the protein dataset of interest, with functional protein domains from a user-specified HMM profile database (Fig. 1c). This database can be the one generated using the IterHMMBuild package of CusProSe, as exemplified in the scheme of Fig. 1a, or, alternatively, any other compatible HMM profile database (.hmm file format). In the second step, annotated proteins are filtered following a set of rules which are also user-determined (Fig. 1c). The rules describe any protein families of interest based on the user-defined specific domain architectures. Features defining each rule include the protein family name, the “mandatory” domains (list of domains the protein must contain) and the “forbidden” domains (optional, list of domain the protein must not contain). All proteins matching those rules are then finally accessible in the ProSeCDA output files directory. The output files include, for each identified protein, a summary in xml format, containing information such as the protein sequence and the boundaries of the conserved domain architectures (.xml), the protein sequence in fasta format (.fa), as well as plots showing a graphical representation of all of the domains that matched to the rules at the pdf format (optional, .pdf). (Figs. 1c, 2b,c). An interactive web page allowing to visualize the ProSeCDA annotation results is also created (index.html file). This web page is illustrated in Fig. 2a.

### Application of CusProSe to the prediction of fungal SMKEs

As an example of application, CusProSe was used to identify major families of SMKEs in fungi. The tools developed were first tested to detect PKS, NRPS, hybrid PKS-NRPS (including NRPS-PKS), and DMATS enzymes, in four species of phylogenetically unrelated plant pathogenic fungi with different host spectra, infection lifestyles and SM repertoires: (i) *Botrytis cinerea* (Leotiomycetes), a necrotrophic pathogen responsible for gray mold on more than 200 dicotyledons including grapevine, (ii) *Colletotrichum higginsianum* (Sordariomycetes), a hemibiotroph which attacks many cultivated plants among Brassicaceae as well as the model plant *Arabidopsis thaliana*, (iii) *Zymoseptoria tritici* (Dothideomycetes), a hemibiotroph which causes the most important foliar disease of wheat (“Septoria tritici blotch”) and (iv) *Magnaporthe oryzae* (Sordariomycetes), also a hemibiotroph, responsible for the most important disease of rice worldwide, rice blast^3,4,20–24^.

First, HMM profiles were constructed using *M. oryzae* protein sequences of conserved functional domains (Table 1) characteristic of PKS, NRPS, PKS-NRPS and DMATS enzymes^25^. At this stage three PKS, three NRPS, three hybrid PKS-NRPS and three DMATS were used to seed IterHMMBuild (Supplementary Data S1). HMM profiles were also constructed for type III PKS (t3PKS), using conserved domain sequences of two *M. oryzae* t3PKS enzymes (Supplementary Data S1). These initial domain profile models were then used to screen the *M. oryzae* proteome to identify potential new domains by homology search, to improve the HMM profiles. The database of domain profiles generated by IterHMMBuild was then given as input to ProSeCDA to annotate the *M. oryzae* proteome, together with the rules file. The rules used to define each type of SMKE above cited can be found in the supplementary File S1. This protocol made it possible to detect all PKS, NRPS, PKS-NRPS and DMATS from *M. oryzae*^25^. The enriched HMM domain profile models were subsequently used to screen the *C. higginsianum* proteome, which again led to the identification of all SMKEs and to further enrich the HMM profiles. The same process was applied to *B. cinerea* and to *Z. tritici*. The identified proteins were manually validated at each step of the analysis. Comparison with existing data showed that the results obtained with CusProSe for these four fungi were consistent with previous SMKEs annotations^4,22,24,25^.

**Table 1:** List of PKS, NRPS and DMATS essential functional domains.

Class	Full name	Essential domains
PKS	Polyketide synthase	Acyl transferase (AT)
		Ketoacyl synthase (KS)
		Phosphopantetheine binding domain (PP-binding)
NRPS	Non-ribosomal peptide synthetase	Adenylation (A)
		Condensation (C)
		Phosphopantetheine binding domain (PP-binding)
DMATS	Dimethylallyl tryptophan synthase	Trp_DMAT

### Comparison of CusProSe with existing SMKEs predictors

**Figure 3:**
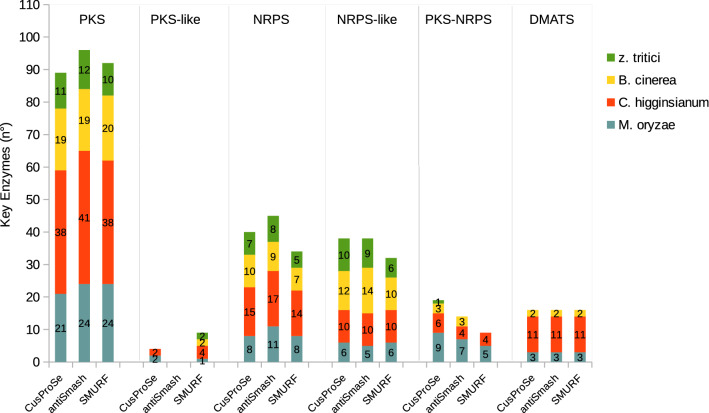
Number of fungal SMKEs identified by CusProSe, antiSMASH and SMURF. The graphic displays the number of proteins identified with CusProSe, antiSMASH and SMURF for PKS and PKS-like, NRPS and NRPS-like, PKS-NRPS (also including NRPS-PKS) and DMATS SMKEs families from *Magnaporthe oryzae* (blue), *Colletotrichum higginsianum* (red)*, Botrytis cinerea* (yellow) *and Zymoseptoria tritici* (green)*.*

**Figure 4:**
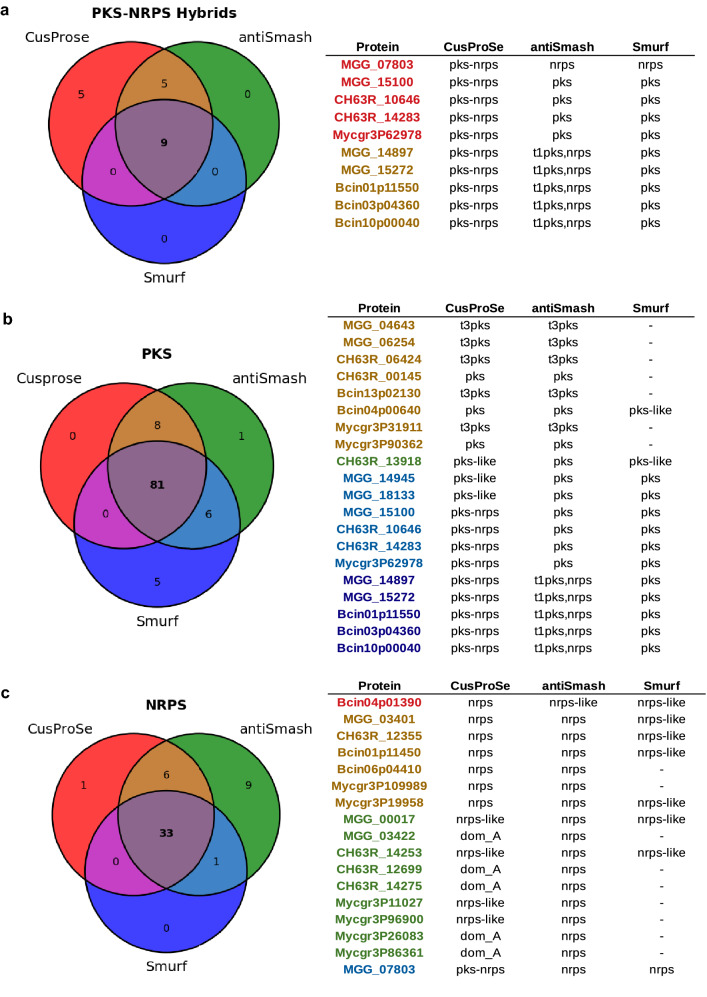
Venn diagrams of SMKEs detected with CusProSe, antiSMASH and SMURF. (**a**) PKS-NRPS*; (**b**) PKS; (**c**) NRPS. CusProSe SMKEs are labeled in red, while antiSMASH and SMURF SMKEs are labeled in green and blue, respectively. Tables on the right for each (**a–c**) panels highlight the proteins differing in their annotation prediction according to the different predictors. The color code is the same as in the Venn diagrams. MGG_ID: *Magnaporthe oryzae*, CH63R_ID: *Colletotrichum higginsianum, BcinID: Botrytis cinerea, Mycgr3PID, Zymoseptoria tritici*. Asterisk, includes NRPS-PKS hybrids.

To assess the performance of CusProSe in predicting SMKEs relative to other predictors, the catalogs of proteins obtained for each fungus were compared to those obtained with antiSMASH^18^ and SMURF^16^. The total numbers of SMKEs detected with the three predictors are shown in Fig. 3. The list of all proteins identified is available in supplementary data (File S3). Comparisons of the results obtained with the three software are also presented in Venn diagrams and tables of Fig. 4. As shown in both Figs. 3 and 4, the effectiveness of CusProSe, antiSMASH and SMURF software varies according to the families of SMKEs. For DMATS, all members of this family were detected by the three software (Fig. 3). In contrast, differences were observed between the software for both the number of sequences recovered and the number of sequences correctly assigned, regarding PKS, NRPS and the PKS-NRPS hybrid enzymes. For PKS-NRPS enzymes, a significant number of false negatives (FN) were observed with antiSMASH and SMURF (Figs. 3 and 4). These missed enzymes were wrongly annotated as NRPS or PKS. For instance, the *M. oryzae* NRPS-PKS hybrid MGG_07803 enzyme, involved in tenuazonic acid biosynthesis^26^, was assigned as NRPS by both antiSMASH and SMURF, while it was correctly detected as a hybrid enzyme by CusProSe. Overall, antiSMASH correctly assigned 14 of the 19 hybrid enzymes (74%), whereas only 9 were identified by SMURF (47%)*.* In contrast, CusProSe successfully detected all the 19 PKS-NRPS/NRPS-PKS hybrids in the four fungal genomes analyzed^4,22,24,25^.

As concerns the total number of PKS, 89 proteins were detected with CusProSe compared to 96 and 92 with antiSMASH and SMURF, respectively. As discussed above, some of the SMKEs detected as PKS by antiSMASH and SMURF are actually PKS-NRPS hybrids and can therefore be considered false positives (FP). Among the seven antiSMASH additional PKS, four were in reality PKS-NRPS hybrids (Fig. 4) attested by the presence of NRPS domains in addition to the PKS module^4,25^. The three other antiSMASH additional PKS were identified as PKS-like by CusProSe. A careful examination of these three proteins revealed that the PP-binding domain, one of the three essential domains of PKS enzymes, is absent from these protein sequences. We made the choice, in our rules file, of considering as PKS only enzymes with all three essential functional domains KS, AT and PP-binding. Those proteins with one missing domain were therefore classified as PKS-like enzymes by CusProSe (Fig. 3). The same holds true for the NRPS enzymes. Regarding SMURF, nine predicted PKS were found to be hybrid PKS-NRPS enzymes by CusProSe. SMURF also missed the five PKS belonging to the type III PKSs (t3PKS), unlike CusProSe and antiSMASH which both have specific profiles / rules for these PKS enzymes.

For NRPS, 40 proteins were detected by CusProSe compared to 45 and 34 by antiSMASH and SMURF, respectively (Fig. 3). The different annotation predictions are illustrated in Fig. 4c. Thirty-three proteins were identified as NRPS by the three predictors, but for 17 cases differences were observed. Regarding the six proteins annotated as NRPS by CusProSe and antiSMASH only, two were missed by SMURF, whereas the four others were annotated by SMURF as “NRPS-like”. Nine proteins were annotated by antiSMASH only as NRPS. From these, two were annotated by CusProSe and SMURF as NRPS-like, whereas the seven others were identifed by CusProSe as NRPS-like (they missed one of the 3 essential domains, Table 1) or “dom_A” (only an isolated adenylation domain was detected). In contrast, one protein was annotated as NRPS by CusProSe alone, being classified as “NRPS-like” by both antiSMASH and SMURF. Finally, as discussed previously, one SMKE detected as NRPS by antiSMASH and SMURF is in fact a hybrid enzyme (NRPS-PKS)^26^, as annotated by CusProSe.

### Identification of Terpene synthase family enzymes

CusProSe was used to improve the detection of Terpene synthases (TS, also referred to as TC or Terpene Cyclases)^27^. TS are SMKEs involved in the biosynthesis of terpenoids, which are among the most structurally and functionally diverse natural compounds^28,29^. They are synthesized in various organisms such as plants, bacteria and fungi^30,31^. The TS are highly variable both in the type of their functional protein domains and in their protein sequences, as compared to other SMKEs^32,33^. This particularity renders more difficult their detection by bioinformatic methods. As a consequence, TS analysis was not considered in the SMURF software^16^, whereas antiSMASH does not distinguish between the different families of TS. The lack of a good TS detection method by currently available tools was therefore a challenging issue.

**Figure 5:**
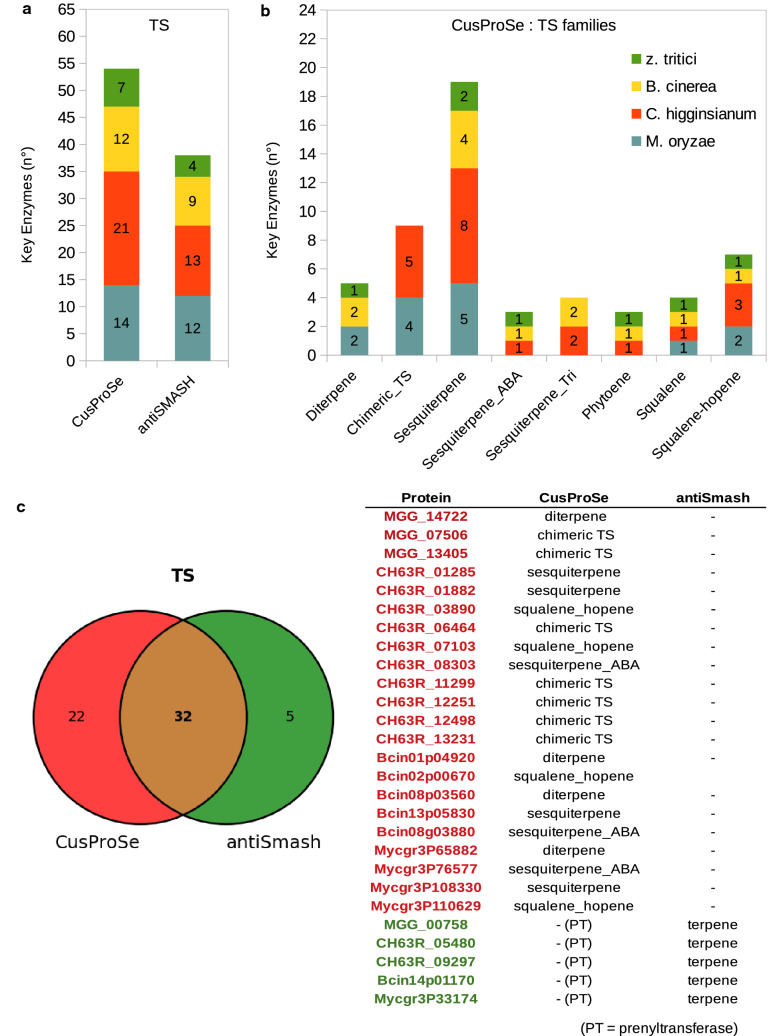
Identification of TS by CusProSe in four fungal genomes and comparison with antiSMASH. (**a**) Number of TS identified by CusProSe and antiSMASH in *Magnaporthe oryzae*, *Colletotrichum higginsianum, Botrytis cinerea, and Zymoseptoria tritici*. (**b**) Number of TS identified with CusProSe for each sub-family in the four fungal genomes (**c**) Venn diagram representing the efficiency of CusProSe for detection of TS compared to antiSMASH. The number of CusProSe annotated TS enzymes are shown in red in the Venn diagrams, while the number of TS detected using antiSMASH are shown in green. On the right, proteins differing in their annotation prediction according to predictors.

HMM profiles were constructed separately for five different families of TS, including sesquiterpenes, diterpenes, phytoenes, squalenes, and chimeric TS. These last enzymes are bi-functional proteins presenting both TS and prenyltransferase domains^30^. Specific profiles for sub-families of sesquiterpene synthases, which are the most abundant fungal TS enzymes, were also built. The rules file was enriched to include information on the different TS domain architectures of each sub-family (supplementary File S2). We started from a small number of well-defined fungal TS protein sequences from each of the different TS enzyme sub-families. The set included biochemically characterized proteins and manually annotated and reviewed sequences from the UniprotKB/Swiss-Prot section of the Uniprot knowledgebase^34–41^ (Data S4). HMM profile models for the different TS families were constructed with IterHMMBuild and used to screen the *M. oryzae, C. higginsianum, B. cinerea* and *Z. tritici* proteomes*.* As the SMURF algorithm does not include TS detection, we compared CusProSe predictions to those of antiSMASH only. As can be seen in the data presented in Fig. 5, our profiles and rules lead to a more precise classification of TS. Indeed, we were able to identify the sub-families of TS present in the four different fungi, with additional TS enzymes found relative to the antiSMASH predictions (Fig. 5a,b). CusProSe particularly outperforms antiSMASH for the chimeric TS, but also performed better for diterpene synthases, sesquiterpene synthases, and squalane-hopene synthases (Fig. 5c, see also Fig. S2). In addition, CusProSe avoids false positives such as prenyltransferases (PT), that are implicated in terpenoids biosynthetic pathways but are not TS enzymes per se^42^. These proteins were classified as TS by antiSMASH (5 FP) (Fig. 5c). Parallel phylogenetic analyses confirmed our classification of TS into these sub-families (supplementary Fig. 3A,B).

### Application of CusProSe to other fungal genomes

To further evaluate the efficiency of our HMM profiles and rules in identifying fungal SMKEs, they were both used to mine other unrelated fungal genomes. We chose representative fungal species with well-annotated genomes from different taxonomic classes : *Aspergillus nidulans* and *Aspergillus niger* from Eurotiomycetes, *Fusarium fujikuroi* and *Fusarium graminearum* from Sordariomycetes, and *Leptosphaeria maculans* from Dothideomycetes^43–46^. The SMKEs identified for each fungus using the custom HMM profiles and ProSeCDA annotation rules were carefully analyzed and compared to previously published annotations^43,44,47,48^. The number and lists of all SMKEs are displayed respectively in Fig. S4 and File S5. A comparison with the results obtained with antiSMASH showed that CusProSe performed particularly better in the identification of the distinct TS enzymes (Figs. S5, S6). Beyond confirming the accuracy of the CusProSe predictions, these novel analyses of fungal genome mining for SMKEs further enriched the fungal-specific HMM profiles of each family, with additional sequences from phylogenetically diverse fungi. These HMM models may be useful to the scientific community interested in fungal SM and we hope they can contribute to identifying other as-yet-unknown KEs.

## Discussion

CusProSe, a novel protein annotation software, is a versatile and flexible tool that can be customized to suit different types of genome/proteome analyses. As an illustration, this software was here used to mine fungal genomes for key enzymes involved in SM.

One of the goals of CusProSe was to improve the accuracy of detection of each type of fungal SMKEs. Our HMM profiles were built exclusively from well-annotated fungal genome sequences already mined for SMKEs. Our main purpose was to carry out a careful analysis of the output of CusProSe including a manual examination of the predicted enzymes. We focused our search on major fungal SMKEs such as PKS, NRPS, hybrid PKS-NRPS, DMATS, and the large family of TS.

CusProSe performance was either similar to, or better than, antiSMASH and SMURF for the identification of fungal SMKEs. This is due to a combination of both specific profiles and strict and carefully defined rules. In particular, CusProSe performed better for PKS-NRPS hybrids. CusProSe also clearly differentiated complete PKS and NRPS from PKS-like or NRPS-like enzymes. Moreover, for TS CusProSe was more sensitive than antiSMASH as it allowed the detection of all TS types and their classification into distinct sub-families, thanks to sub-family specific HMM profiles and rules, avoiding false positives. This is a major novelty and advantage as regards the search for TS enzymes, relatively to the existing predictors. Finally, our data demonstrate that, using HMM profiles built from a few well-annotated and representative proteins, and well-defined and adequate rules, it is possible to accurately identify candidate proteins of each enzyme family with high sensitivity and specificity.

CusProSe has so far been successfully used with genomes from well-known fungal species, representing the Eurotiomycetes, Leotiomycetes, Sordariomycetes and Dothideomycetes classes of the Ascomycota. Different species representing these or other fungal classes may also be mined*.* Our HMMs models may be further enriched by mining additional fungal genomes, including genome sequences from representatives of the Basidiomycota. The database of fungal HMM profiles created within the framework of the present study is available to the community, as well as the file of rules used by ProSeCDA for protein annotation. Each user can either use these tools directly or adapt them/create new ones according to specific needs. The rules file can evolve to take into account new requirements, for example detection of a new protein family or sub-family. This means that a rule for a novel protein family can be added. A rule can also be easily deleted, or modified, in this case by adding a new “mandatory” or “forbidden” protein domain for example. A completely new rules file can also be constructed either manually using the required yaml format or the proposed graphical interface, as specified in the CusProSe Documentation. Importantly, CusProSe is not restricted to the prediction of SMKEs. It is a custom genome mining software package, whose integrating tools can be exploited in the context of any protein family / biological system. Its successful utilization for a fast, easy and accurate prediction of major fungal SMKEs presented here illustrates its interest to a broad community of biologists.

## Methods

### Implementation, system requirements and availability

CusProSe was coded on Python 3, and details on its implementation can be found at the Documentation page https://i2bc.github.io/CusProSe/. The application requires Python version 3.7. The external dependencies are the programs HMMER (version 3.3)^49^, MUSCLE (version 3.8.1551)^50^, and Usearch^51^. CusProSe is freely available. Its source code is distributed at https://github.com/i2bc/CusProSe and updated versions will be accessible there. CusProSe is compatible with all platforms supporting Python, HMMER, and MUSCLE. The user runs the CusProSe tools IterHMMBuild and/or ProSeCDA from the command line. The two packages can be run independently. The main steps of both pipelines are summarized in this “Methods” section. A usage guideline with more detailed information and examples is available at the CusProSe Documentation page (https://i2bc.github.io/CusProSe/).

### IterHMMbuild package

#### Rationale, input and outputs of IterHMMBuild

The CusProSe IterHMMBuild tool allows the user to build an HMM profile representative of (a) seed protein sequence(s) by using a set of other defined protein sequences to iteratively feed the HMM profile (training set). Two inputs are required in a fasta file format: (i) either a fasta file with at least one protein sequence or a directory location where multiple individual fasta files are stored and (ii) a fasta file of protein sequences (dataset), such as a proteome, used to enrich initial protein sequence(s) of interest. There is no limit on protein sequence length, genome size or any other similar constraints for the input files. Input working examples are available in the cusProSe/iterhmmbuild/datas directory of the CusProSe archive (https://github.com/i2bc/CusProSe). The fasta sequences of each seed set are aligned with MUSCLE and the resulting multiple sequence alignment is given as input to HMMER (both integrated in the IterHMMBuild software). The HMM profiles are then built for each domain/family with an iterative search protocol (see Sequence enrichment step below). An output directory includes, for each protein/protein domain of interest, the final HMM profile file (domain.hmm), the final sequences file in fasta format used to build the HMM profile (domain_seed.fa), the multiple alignment file of those sequences at Clustal W format (domain_seed.clw), a log file containing a summary of the computation (info.log), and iter_i directories containing files obtained at each iteration cycle (i). More details on the iter_i/ files as well on the usage of IterHMMBuild and its different possibilities and parameters can be found at the IterHMMBuild Usage guideline section of the Documentation file (https://i2bc.github.io/CusProSe/ihb_usage.html).

#### Sequence enrichment step

The HMM profiles initially built are searched against the protein dataset given as input using the hmmsearch command from HMMER, integrated in the software. The purpose is to enrich the HMM profile models. All matching sequences with E-values less than 0.01 (default value) and an expected accuracy per residue of the alignment above or equal to 0.6 (default value) are retrieved. Those sequences are then merged to the initial input sequences. To ensure that sequences are not redundant, Usearch is applied with a threshold identity value of 90%. A new HMM profile is then build and the process is repeated until convergence is reached. The convergence is reached when the number of sequences at iteration i + 1 (Nseq^i+1^) is strictly equal to the number of sequences at iteration i (Nseq^i^). However, because the number of new sequences found after multiple consecutive iterations can be really low, or even negative, a counter is also used to evaluate the convergence status in order to prevent unnecessary iterations (i. e. iterations that will increase the computation time without significantly enrich the number of new sequences). This counter is incremented each time the difference between Nseq^i+1^ and Nseq^i^ is negative or equal to 1 (default value). The convergence is then also reached when the value of this counter is equal to 3 (default value). In the end of the process a last HMM profile is build, the final enriched HMM profile.

#### Building the HMM profile database

An HMM profile database from a set of individual enriched HMM profiles representative of different domain sequences of interest can be generated manually, by concatenation of the profiles, with the command create_hmmdb. This is the case when profiles for each protein domain of interest are constructed separately in different runs, with a single seed domain fasta file given as input at each run. As an alternative the HMM database can also be built automatically when running IterHMMBuild, if a directory with multiple individual fasta files is given as input (see Documentation for further information on the different possibilities of HMM profiles and database constructions).

### ProSeCDA package

#### Rationale, input and outputs of ProSeCDA

The CusProSe ProSeCDA tool allows the user to detect proteins matching specific given domain architectures rules, defined by the user, in any desired dataset of proteins, such as a proteome. Three input files are required: a dataset of proteins in fasta format (dataset.fa), an HMM profile database (database.hm), and a file describing the rules (rules.yaml). As for IterHMMBuild there are no particular input constraints besides the specified file format. Input working examples are available at the cusProSe/prosecda/datas directory of the CusProSe archive (https://github.com/i2bc/CusProSe). The output of ProSeCDA includes (i) a info.log file containing the summary log of the computation run, (ii) an output file of hmmsearch for the protein dataset analyzed (dataset.domtblout), (iii) an interactive web page allowing to visualize the results (index.html), (iv) 3 additional folders containing files read by index.html (css/ js/ and images/) and (v) a directory nammed "Results" containing different output files for rules matching proteins. This directory contains sub-directories for each rule for which proteins have been found, where the user can find, for each identified protein, an individual summary at an xml format, containing information such as the protein sequence and the boundaries of the conserved domain architectures (protein_ID.xml). The protein sequence file in fasta format is also provided for each individual match (protein_ID.fa). Plots at the pdf format (protein_ID.pdf) showing a graphical representation of all of the domains that matched to the rules for a given protein can also be created (optional) and are displayed in the results/ sub-directories. Examples are shown in Fig. 2 of the manuscript. The computation time of an average Prosecda run with default parameters, on a computer with a Intel^®^ Core™ i7-4900MQ CPU @ 2.80 GHz × 8 processor running the Linux operating system, was about 2.4 sec. This time was increased to 2.5 min on average with the plots at the pdf format option.

#### Creation of rules

Rules are written in a specific yaml (Yet Another Markup Language) format (https://yaml.org/). A Graphical User Interface (GUI) accessible by running the create_rules script was implemented to help users in this task (see details in the CusProSe Documentation file). Each rule was defined by different features: Name (protein category/family name), Comment (optional feature; can be used to describe the rule), Mandatory list (list of domain names the protein must contain), and Forbidden list (optional list of domain names the protein must not contain).

#### Annotation step: selection of matching domains

The annotation step of ProSeCDA assigns domains from the user-defined HMM profile database to matching sequences from a protein dataset of interest. The annotation procedure uses hmmsearch from HMMER to search for each of the HMM domain profile present in the user-defined rules file (see Creation of rules above) against the protein dataset. All matching sequences with an E-value less than 0.01 (default value) and an expected accuracy per residue of the alignment above or equal to 0.6 (default value) are then retrieved. The E-value and accuracy can be changed by the user. Both conditional and independent E-values from HMMER are evaluated.

To resolve overlapping domains (we considered that two domains are overlapping if at least 40% of the shortest domain sequence overlap with the other domain), an approach similar to the heaviest weighted clique-finding method described by Yeats and collaborators^52^ was used. When multiple matching domains are found for a protein sequence and some of those domains overlap, all possible domain architectures defined by a set of non-overlapping domains are identified, with each domain being assigned a score corresponding to -log(E-value). An alternative score is also used in case the E-value associated with a match is equal to 0.0. In that case, the bit score from HMMER is assigned to each domain instead of the -log(E-value) which cannot be computed. The protein is then assigned to the most-likely domain architecture which is defined as the combination of non-overlapping domains that gives the highest total score.

#### Filtering step

The filtering step searches in all previously annotated proteins the domain architectures matching those described in the set of rules defined by the user. A match with a user-defined family is valid for a protein if its most-likely domain architecture fits the mandatory domains and if no forbidden domains are present. Moreover, if an E-value threshold is specified in the rules for a given mandatory domain, this domain must match with an E-value at least below this threshold.

#### Annotation of SMKEs with CusProSe and comparison with antiSMASH and SMURF

The test sets used to evaluate CusProSe predictions were composed of protein sequences from known protein families from manually-anotated and revised fungal genomes and also from experimentally characterized proteins. The sequences are available in the Supplementary material (Folders Data S1 and Data S2). The initial training set was the genome of the fungus Magnaporthe oryzae (v. 8, Broad Institute) used to create the first hmm models. These were latter enriched by screening other fungal genomes. The enriched hmm models were then tested in a second round of analysis on additional well annotated fungi representive of different species and classes. The same fungal genomes mined with our tool (same version coming from the same public database) were given as input to antiSMASH^18^ and SMURF^8^ software (FASTA + GFF3 files as recommended) and the programs were run using their respective integrated profiles and rules.

#### Phylogenetic analysis

All sequences were aligned with muscle 3.8 and maximum likelihood trees were made with phyml 3.3 (https://ngphylogeny.fr). The chosen substitution model is LG with an initial BioNJ tree. The bootstrap values obtained after 100 replicates are shown on the tree branches. Trees were generated using Interative Tree of Life (https://itol.embl.de).

#### Fungal genomes and proteomes

Genomes /proteomes used in this work are listed in Table S2 and were downloaded from their respective databases as indicated.

## Supplementary Information

Supplementary Information.

## Data Availability

All data underlying the findings are fully available without restriction. All relevant data are within the paper and its Supporting Files.

## Abbreviations

SM: Secondary metabolism
SMKE: Secondary metabolism key enzymes
PKS: Polyketide synthases
NRPS: Non-ribosomal peptide synthetases
PKS-NRPS: PKS-NRPS or NRPS-PKS hybrid enzymes
DMATS: Dimethylallyl tryptophan synthases
TS: Terpene synthases
ABA: Abscisic acid
Tri: Trichodiene
PT: Prenyltransferases
*M. oryzae*: *Magnaporthe oryzae*
*C. higginsianum*: *Colletotrichum higginsianum*
*B. cinerea*: *Botrytis cinerea*
*Z. tritici*: *Zymoseptoria tritici*
*A. nidulans*: *Aspergillus nidulans*
*A. niger*: *Aspergillus niger*
*F. fujikuroi*: *Fusarium fujikuroi*
*F. graminearum*: *Fusarium graminearum*
*L. maculans*: *Leptosphaeria maculans*
