# Supplementary material for: CusProSe: a customizable protein annotation software with an application to the prediction of fungal secondary metabolism genes
Source: Sci Rep. 2023 Jan 25;13:1417. doi: 10.1038/s41598-023-27813-y (PMC9876896; doi:10.1038/s41598-023-27813-y)
Supplement: Supplementary file 1 — Supplementary Information. [file 41598_2023_27813_MOESM1_ESM.zip › Supplementary_Information_Oliveira/Supplementary_Figures_Oliveira.pdf]

Supplementary Information

CusProSe : A customized protein annotation software applied to fungal specialized metabolism genes

Leonor Oliveira[1*], Nicolas Chevrolier[1,2] , Jean-Fellix Dallery[3], Richard O'Connell[3], Marc-Henri Lebrun[3], Muriel Viaud[3] and Olivier Lespinet[1]

[1]Université Paris-Saclay, CEA, CNRS, Institute for Integrative Biology of the Cell (I2BC), 91198, Gif-sur-Yvette, France

[2]Current Address : Orphanet - INSERM, US14, Plateforme des maladies rares, Paris, France

[3]Université Paris-Saclay, INRAE, UR BIOGER, 78850 Thiverval-Grignon, France

[*]Correponding Author (e-mail: leonor.oliveira@i2bc.paris-saclay.fr)

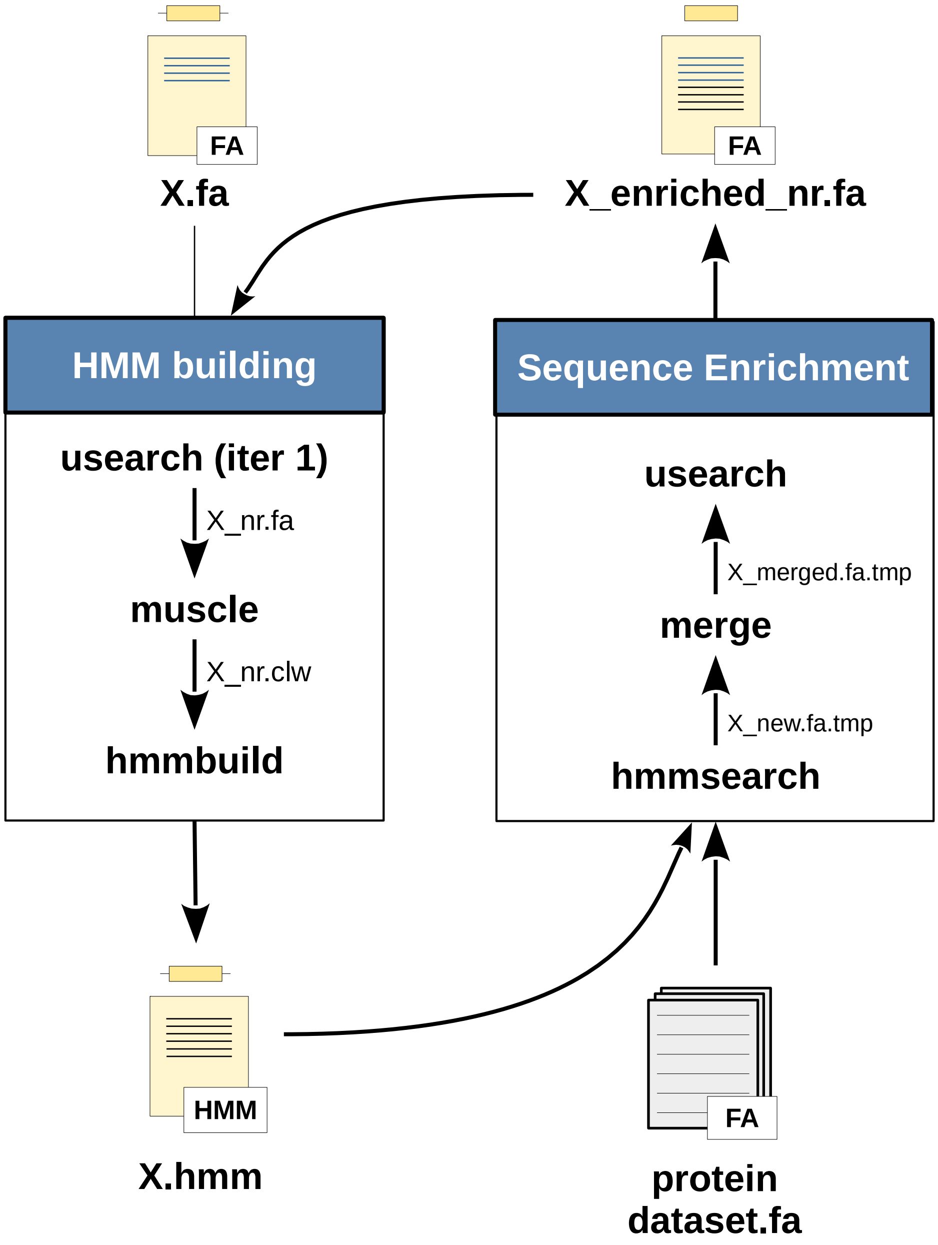

Figure S1. Detailed pipeline of the IterHMMBuild enrichment procedure.

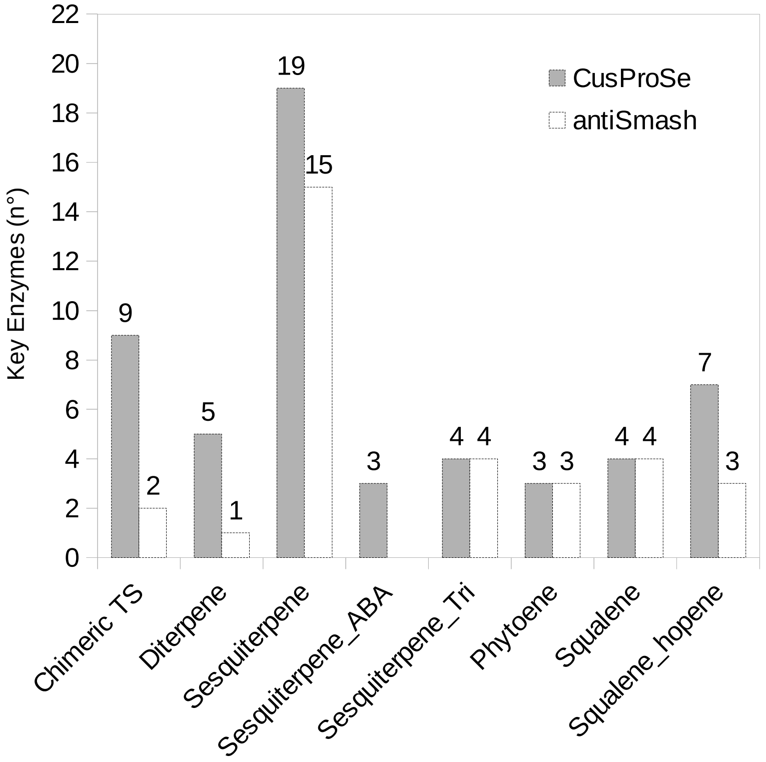

Protein	CusProSe	antiSmash
MGG_14722	diterpene	-
MGG_07506	chimeric TS	-
MGG_13405	chimeric TS	-
CH63R_01285	sesquiterpene	-
CH63R_01882	sesquiterpene	-
CH63R_03890	squalene_hopene	-
CH63R_06464	chimeric TS	-
CH63R_07103	squalene_hopene	-
CH63R_08303	sesquiterpene_ABA	-
CH63R_11299	chimeric TS	-
CH63R_12251	chimeric TS	-
CH63R_12498	chimeric TS	-
CH63R_13231	chimeric TS	-
Bcin01p04920	diterpene	-
Bcin02p00670	squalene_hopene	-
Bcin08p03560	diterpene	-
Bcin13p05830	sesquiterpene	-
Bcin08g03880	sesquiterpene_ABA	-
Mycgr3P65882	diterpene	-
Mycgr3P76577	sesquiterpene_ABA	-
Mycgr3P108330	sesquiterpene	-
Mycgr3P110629	squalene_hopene	-
MGG_09239	squalene	terpene
MGG_00792	squalene_hopene	terpene
MGG_01628	sesquiterpene	terpene
MGG_01701	chimeric TS	terpene
MGG_01949	diterpene	terpene
MGG_03432	chimeric TS	terpene
MGG_03833	sesquiterpene	terpene
MGG_04631	sesquiterpene	terpene
MGG_10516	sesquiterpene	terpene
MGG_10671	sesquiterpene	terpene
MGG_11702	squalene_hopene	terpene
CH63R_06219	squalene	terpene
CH63R_07462	phytoene	terpene
CH63R_11561	squalene_hopene	terpene
CH63R_05405	sesquiterpene_Tri	terpene
CH63R_05999	sesquiterpene_Tri	terpene
CH63R_06527	sesquiterpene	terpene
CH63R_08252	sesquiterpene	terpene
CH63R_11027	sesquiterpene	terpene
CH63R_11724	sesquiterpene	terpene
CH63R_12275	sesquiterpene	terpene
CH63R_12288	sesquiterpene	terpene
Bcin01p03520	sesquiterpene	terpene
Bcin01p04560	phytoene	terpene
Bcin04p03550	sesquiterpene	terpene
Bcin06p02400	squalene	terpene
Bcin08p02350	sesquiterpene_Tri	terpene
Bcin11p06510	sesquiterpene_Tri	terpene
Bcin12p06390	sesquiterpene	terpene
Mycgr3P51545	phytoene	terpene
Mycgr3P65850	squalene	terpene
Mycgr3P6941	sesquiterpene	terpene
MGG_00758	- (PT)	terpene
CH63R_05480	- (PT)	terpene
CH63R_09297	- (PT)	terpene
Bcin14p01170	- (PT)	terpene
Mycgr3P3174	- (PT)	terpene

(PT = prenyltransferase)

Figure S2. TS identified with CusProSe compared to antiSMASH for Magnoporthe oryzae (MGG_ID), Colletotrichum higginsianum (CH63R_ID), Botrytis cinerea (BcinID) and Zymoseptoria tritici (Mycgr3PID). ID colour code: red, identified only by CusProSe; green, identified only by antiSMASH; brown, identified both by CusProSe and antiSMASH. PT, prenyltransferase.

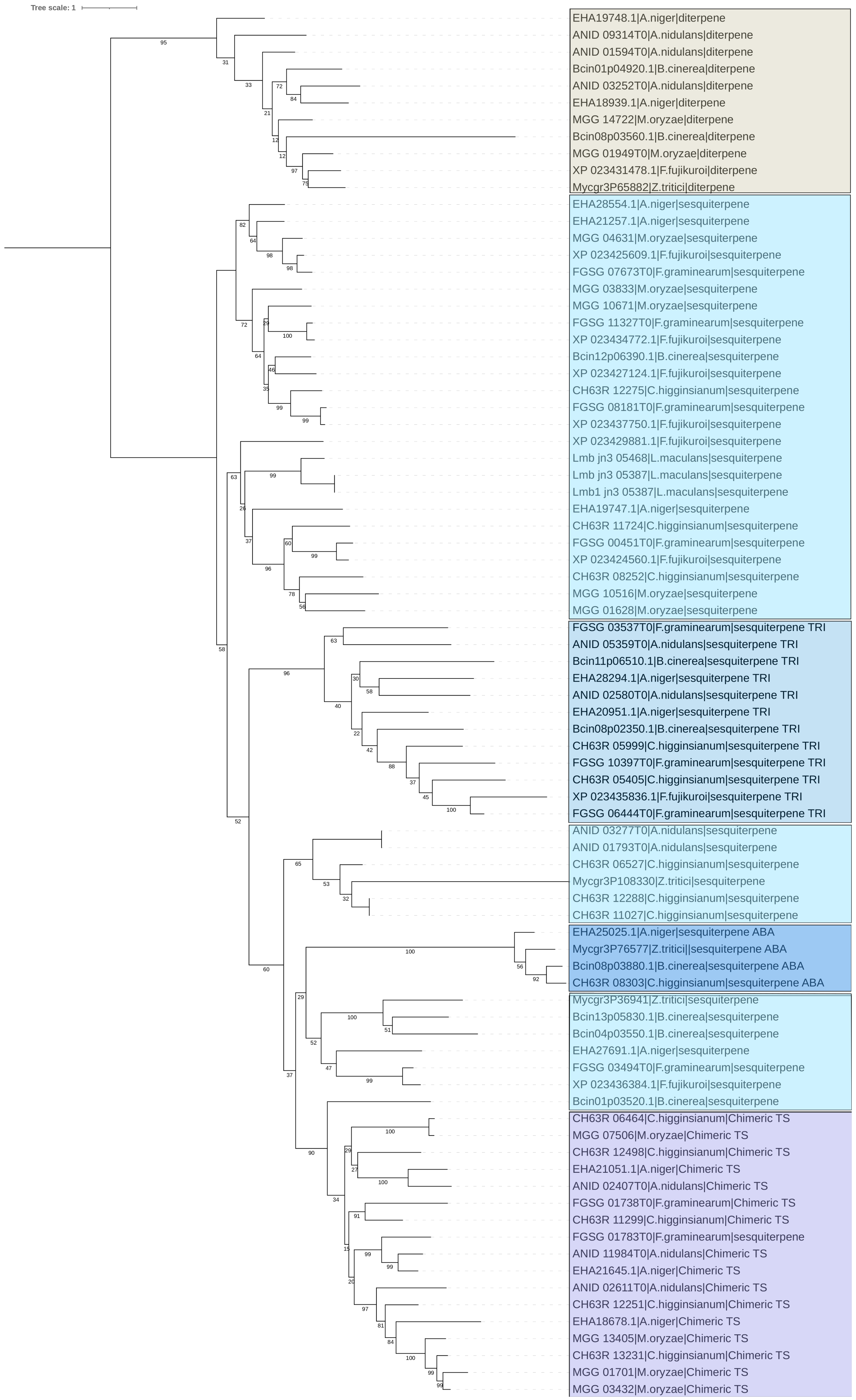

Figure S3A. Phylogenetic analysis of fungal TS identified by CusProSe (diterpenes, sesquiterpenes and chimeric-TS).

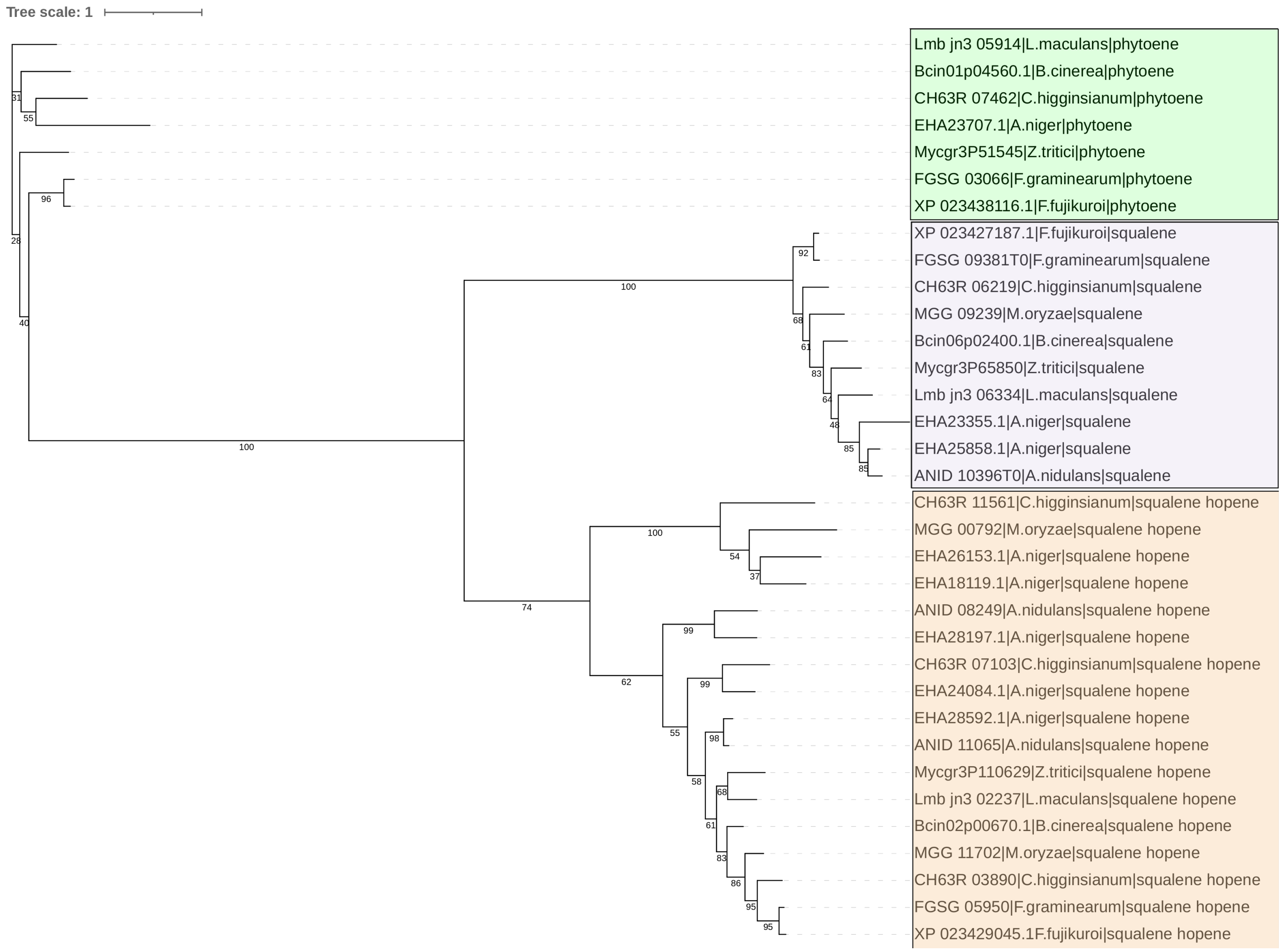

Figure S3B. Phylogenetic analysis of fungal TS identified by CusProSe (phytoene, squalene et squalene-hopene syntases).

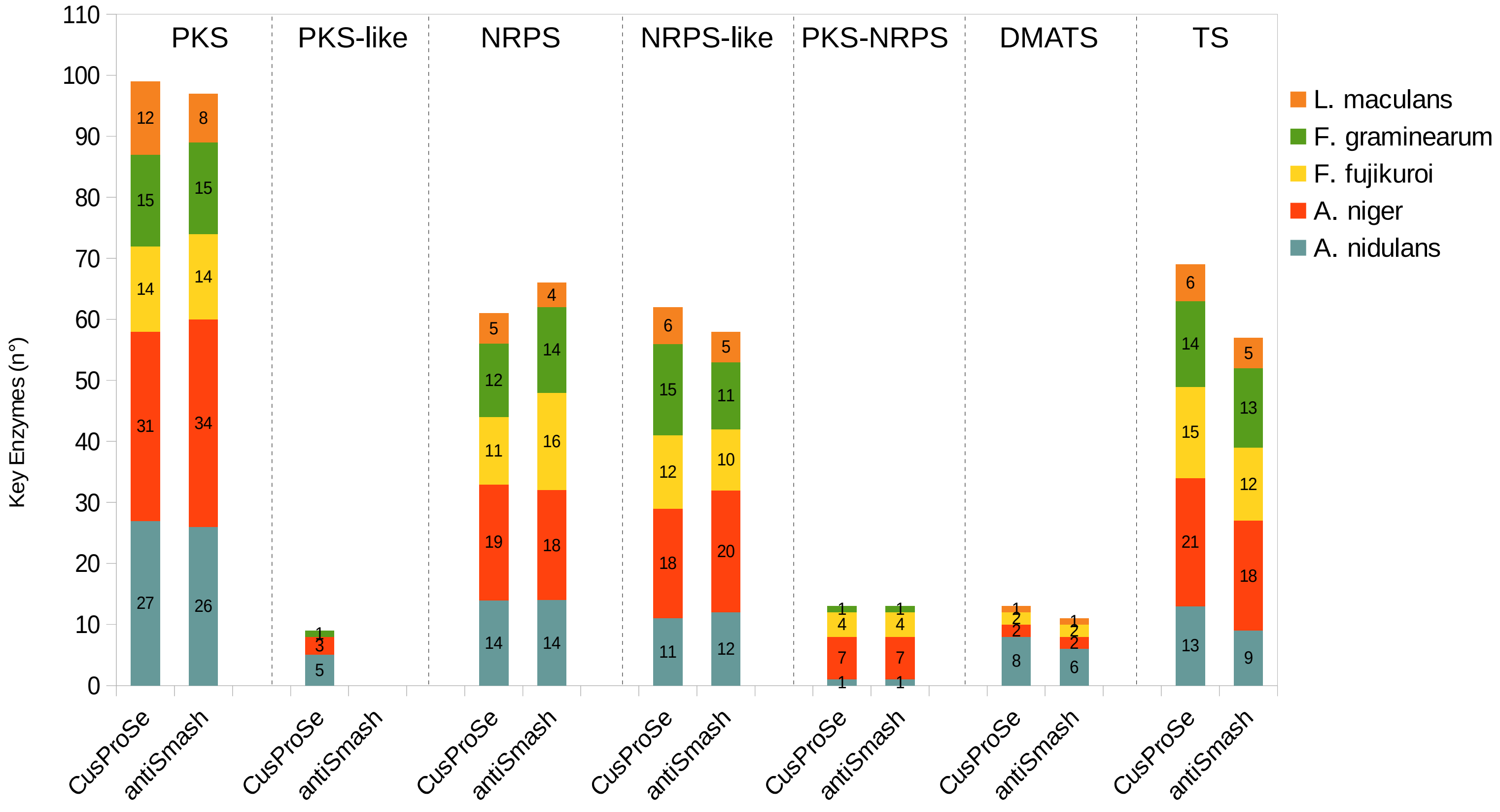

Figure S4. Number of SMKEs identified by CusProSe and antiSMASH in Aspergillus, Fusarium and Leptosphaeria fungi species. *A. nidulans, Aspergillus nidulans; A. niger, Aspergillus niger; F. graminearum, Fusarium graminearum; F. fujikuroi, Fusarium fujikuroi; L. maculans, Leptosphaeria maculans.*

a

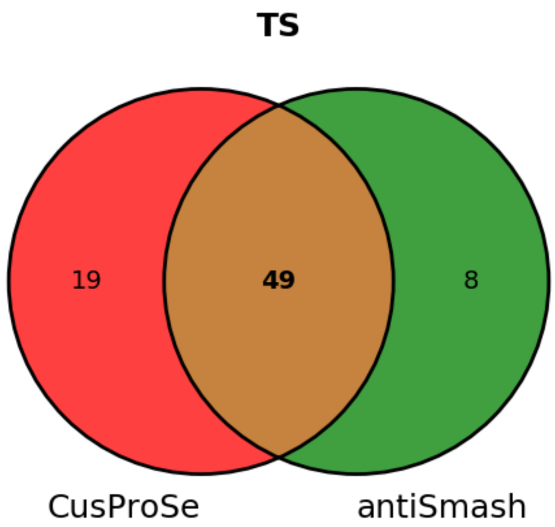

Protein	CusProSe	antiSmash
ANID_01594	diterpene	-
ANID_03252	diterpene	-
ANID_09314	diterpene	-
ANID_01793	sesquiterpene	-
ANID_02407	chimeric_TS	-
ANID_02611	chimeric_TS	-
ANID_11455	sesquiterpene	-
ANID_11984	chimeric_TS	-
EHA18939.1	diterpene	-
EHA19748.1	diterpene	-
EHA21051.1	chimeric_TS	-
EHA21645.1	chimeric_TS	-
EHA24084.1	squalene_hopene	-
EHA25025.1	sesquiterpene_ABA	-
EHA28197.1	squalene_hopene	-
FGSG_01738T0	chimeric_TS	-
FGSG_05950T0	squalene_hopene	-
XP_023429045.1	squalene_hopene	-
Lmb_jn3_11564	sesquiterpene_ABA	-
ANID_00654	- (PT)	terpene
EHA18172.1	- (PT)	terpene
EHA19457.1	- (PT)	terpene
EHA20967.1	- (PT)	terpene
EHA20968.1	- (PT)	terpene
FGSG_04591T0	- (PT)	terpene
FGSG_10097T0	- (PT)	terpene
XP_023430643.1	- (PT)	terpene

(PT = prenyltransferase)

b

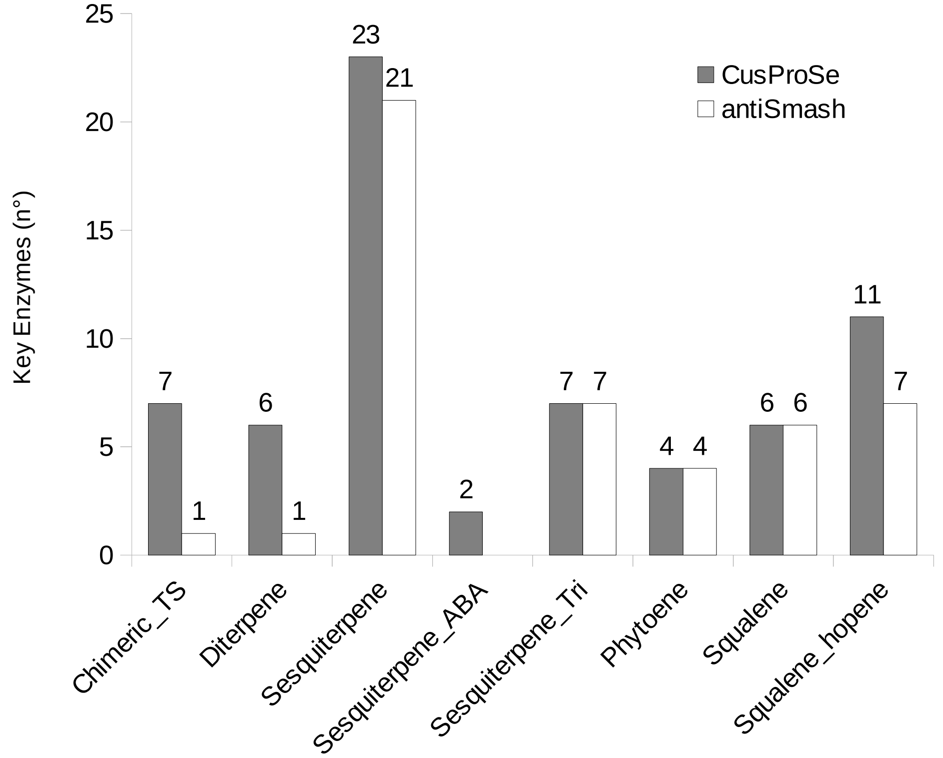

Figure S5. Classification of TS sub-families by CusProSe and comparison with antiSMASH, in Aspergillus, Fusarium and Lepthosphaeria fungi species. A) Venn diagrams of TS detected with CusProSe and antiSMASH. Colour code: red, identified only by CusProSe; green, identified only by antiSMASH; brown, identified both by CusProSe and antiSmash. ANID_ID: *Aspergillus nidulans*, EHAID: *Aspergillus niger;* FGSG_ID: *Fusarium graminearum;* XP_ID, *Fusarium fujikuroi;* Lmb_jn3_ID: *Leptosphaeria maculans*. B) Number of TS detected for each CusProSe defined sub-familly, by CusproSe and antiSMASH (these proteins are classified as terpenes by antiSMASH).

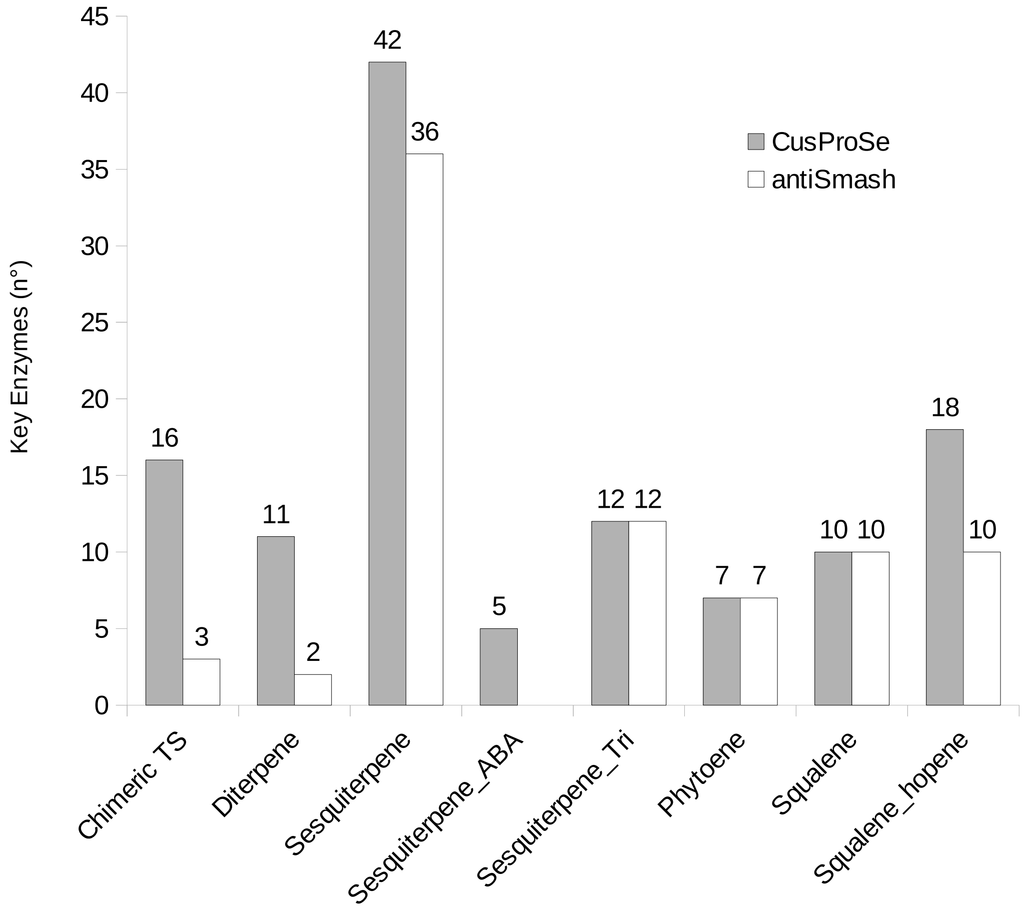

Figure S6. Identification of TS by CusProSe in nine fungal genomes and comparison with antiSMASH. The enzymes are classified as terpenes by antiSMASH. The numbers relate to all the fungi analyzed during this work (*Magnaporthe oryzae*, *Colletotrichum higginsianum*, *Botrytis cinerea*, *Zymoseptoria tritici*, *Aspergillus nidulans*, *Aspergillus niger*, *Fusarium fujikuroi*, *Fusarium graminearum* and *Leptosphaeria maculans*).

Table S2. List of fungal genomes used in the present work

Fungi	Source	Proteome file
Magnaporthe oryzae	Broad Institute	mgg_70-15_8_proteins.fasta
Collectotrichum higginsianum	Joint Genome Institute	Colhig2_GeneCatalog_proteins_20170214.aa.fasta
Botrytis cinerea	Ensembl Genomes	Botrytis_cinerea.ASM83294v1.pep.all.fa
Zymoseptoria tritici	Joint Genome Institute	Zymoseptoria_tritici.MG2.pep.all.fa
Aspergillus nidulans	NCBI	aspergillus_nidulans_fgsc_a4_1_proteins.fasta
Aspergillus niger	NCBI	GCA_000230395.2_ASPNI_v3.0_gbank_protein.faa
Fusarium fujikuroi	NCBI	GCF_900079805.1_Fusarium_fujikuroi_IMI58289_V2_protein.faa
Fusarium graminearum	NCBI	fusarium_graminearum_ph-1_3_proteins.fasta
Leptosphaeria maculans	Fudal I., personnal communication	leptosphaeria_maculans_gsc_gmove_lmb_jn3.pep.fa
